# Total Pancreatectomy with Islet Autotransplantation for the Ampullary Cancer. A Case Report

**DOI:** 10.1007/s12029-017-0029-4

**Published:** 2017-11-21

**Authors:** Omid Savari, Karolina Golab, Julia Solomina, Evelyn Konsur, Kamil Cieply, Sabarinathan Ramachandran, Zehra Tekin, Lindsay Schenck, Sharon S. Zhang, Martin Tibudan, Mitchell C. Posner, Piotr Witkowski

**Affiliations:** 1 Department of Surgery, The University of Chicago, Chicago, IL USA; 2 Department of Pathology, The University of Chicago, Chicago, IL USA

## Introduction

Total pancreatectomy (TP) leads to pancreatic exocrine deficiency and poorly controlled diabetes despite insulin treatment [1]. Simultaneous islet autotransplantation (IATx) can prevent diabetes in 30–40% of patients and improve glycemic control with insulin supplementation in an additional 30% of individuals leading to improvement in quality of life in the majority of patients [2]. Total pancreatectomy with islet transplantation (TPIAT) has been offered mostly as a last resort procedure to highly selected patients with chronic or recurrent acute pancreatitis and intractable pain despite medical, endoscopic, and other conventional surgical interventions. Although patients with advanced benign tumors who require TP also may receive IATx, application of TPIAT in the setting of pancreatic malignancy remains controversial due to the risk of contamination of the islet prep with tumor cells and possible dissemination of malignancy [1]. Nevertheless, encouraging results in single cases of IATx have been reported in those patients with pancreatic cancer, who required TP due to complications after initial Whipple procedure [3–7].

Here we present for the first time, a case report of a patient with a small, early-stage ampullary cancer without lymph node involvement, who developed disseminated neoplastic disease 6 months after TPIAT.

## Method

A 54-year-old male presented with abdominal pain, jaundice, 15-pound weight loss, and pruritus. Initial tests revealed elevated alkaline phosphatase and total bilirubin (1.9 mg%), common bile duct (CBD) dilatation of 14 mm, and a rim-enhancing lesion in the periampullary region measuring 8–11 mm on magnetic resonance cholangiopancreatography (MRCP). Endoscopic ultrasound/endoscopic retrograde cholangiopancreatography (EUS/ERCP) found a 10 × 10 mm mass in the ampulla and 6 × 5 mm peripancreatic lymphadenopathy. A stent was placed in the common bile duct and pancreatic duct to decompress the bile tree and pancreas. Fine needle aspiration (FNA) of the ampullary mass was non-diagnostic for cancer; however, brushing from the CBD structure showed atypical cells suspicious for malignancy. The ERCP procedure was complicated by severe acute necrotizing pancreatitis that required intensive care unit (ICU) treatment, multiple drainage procedures, and prolonged hospitalization. Six months after diagnosis and recovery from this acute phase, the patient underwent a total pancreatectomy to address the ampullary cancer in the setting of persistent abdominal pain with splenic vein thrombosis, pseudocyst, and abscess formation in the pancreatic tail. Due to the small size and isolated location of the primary lesion in the head of the pancreas, the patient was offered islet isolation from the tail of the pancreas and islet autotransplantation in order to improve glucose control and quality of life after the surgery. Prior to admission, his glucose levels were within normal range, but became elevated during pancreatitis (200–250 mg%).

### Total Pancreatectomy

**Fig. 1:**
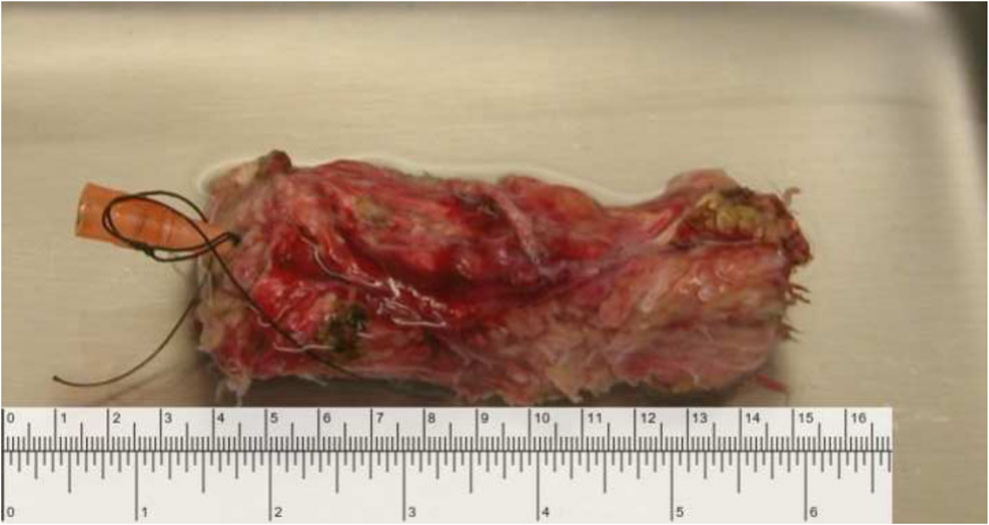
Excised pancreas with angiocatheter inserted into pancreatic duct for islet isolation

There was no evidence of metastatic disease noted during a thorough exploration at the time of surgery. An intraoperative wedge biopsy of the liver segment 4 nodule revealed a bile duct hamartoma. Significant scarring and inflammation of the mesocolon and colon due to pancreatic necrosis and inflammation compromised the blood supply to the colon leading to a near total colectomy. After division of the head and neck of the pancreas, the whole head of pancreas with duodenum and distal common bile duct were sent to pathology for frozen sections. All margins were cancer-free including the neck of the pancreas. Completion pancreatectomy with splenectomy was followed by hepaticojejunostomy, gastrojejunostomy, and anastomosis of ileum to the descending colon. The distal pancreas was dissected from surrounding tissue on the back table. An additional 1 cm of the pancreas neck was resected; the distal pancreatic duct was cannulated with a 14-gage angiocath, while the remaining part of the pancreas sent to our islet isolation facility in cold preservation solution (SPS-1, Organ Recovery System Inc., Chicago, IL) (Fig. 1).

### Islet Isolation and Transplantation

**Fig. 2:**
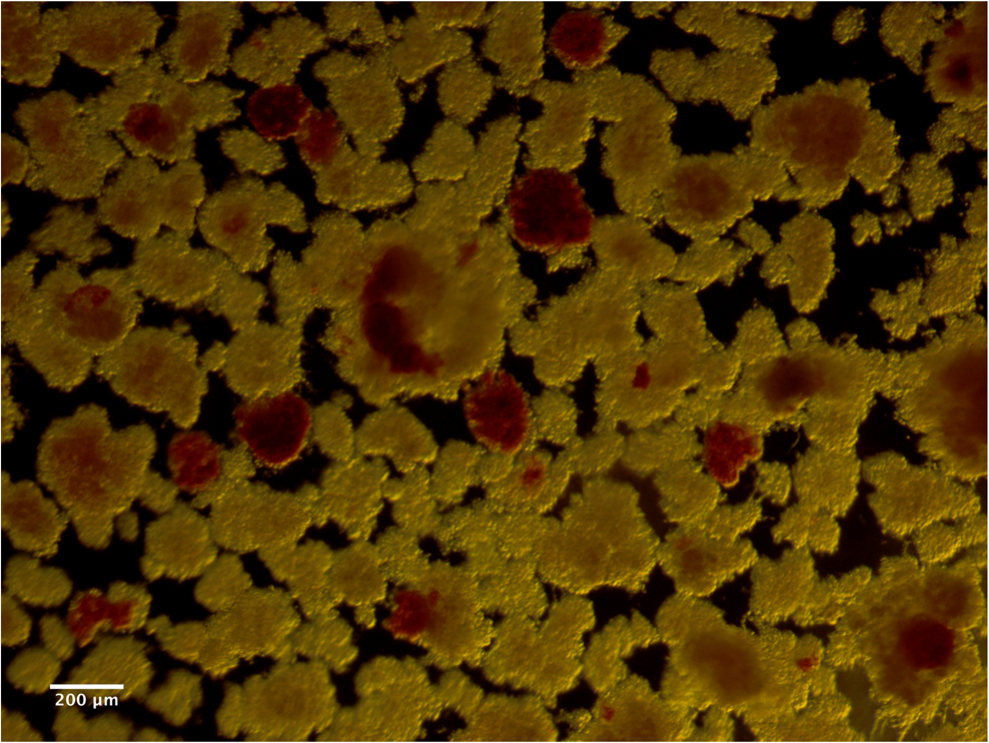
Isolated islets (red) among acinar tissue (brown)

Islets were isolated in the University of Chicago Good Manufacturing Practice (GMP) facility according to the Ricordi method. Cold ischemia was 35 min. The pancreas was perfused with cold collagenase (Liberase, Roche, Indianapolis, IN, USA) for 12 min and then digested in warm temperature for 29 mins. We obtained 64,348 islet equivalents (IEQ) (1205 IEQ/kg of patient body weight) after digestion of 80% of pancreatic tissue (21.9 g out of initial 26.7 g) (Fig. 2).

Islet purification was not applied due to the low pellet volume of 6 ml. The viability based on fluorescein diacetate/propidium iodide staining was 86%. Gram stain and endotoxin were negative. The islets were suspended in the transplantation solution (Mediatech, Manassas, VA, USA) with human albumin and 3700 units of heparin, then infused via 14G angiocatheter into the portal vein without complications just before completion of the entire surgical procedure.

## Results

The patient’s postoperative course was complicated by gastrointestinal (GI) bleeding secondary to an ulcer at the anastomotic site on postoperative day 10, treated endoscopically. The patient was discharged 4 days later with fentanyl patch and hydrocodone/acetaminophen. He was re-admitted 1 week later due to poor oral intake, weight loss, and dehydration. He was discharged home soon after, receiving total parenteral nutrition (TPN).

**Fig. 3:**
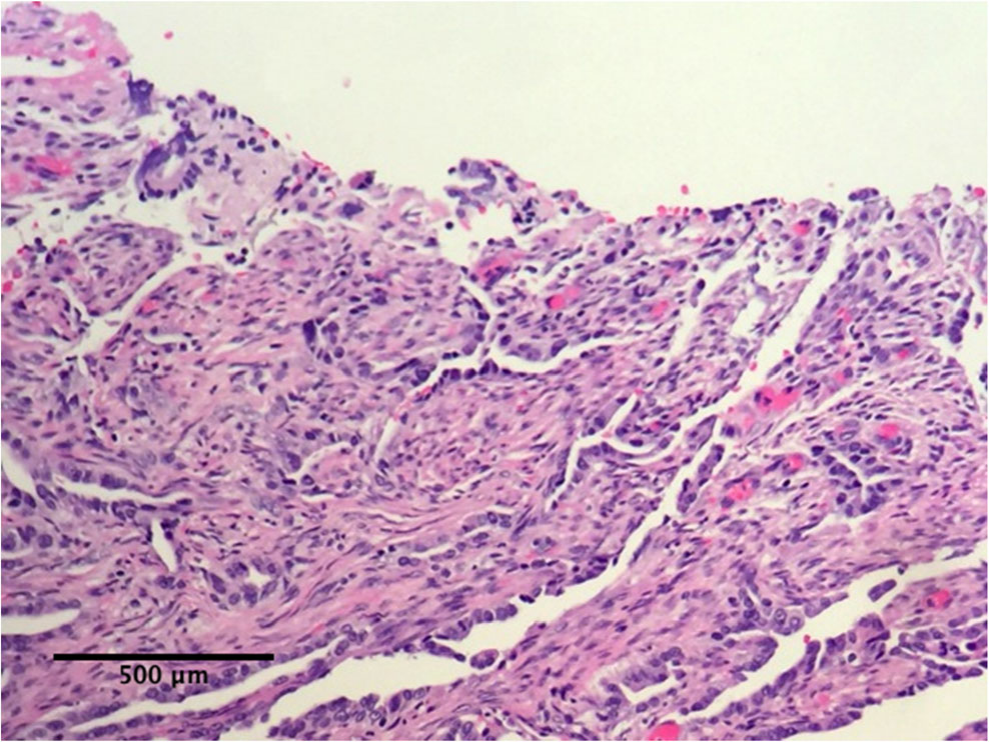
Invasive ampullary adenocarcinoma, moderately differentiated

Postoperatively, and through his entire follow-up, the patient required insulin injections of approximately 30–40 units a day. Random serum c-peptide was 0.05 pmol/ml on day 7 and 0.11 pmol/ml on day 40, which elevated to 0.17 pmol/ml 5 months after the surgery (Fig. 3). At the same time, the patient was not compliant with insulin therapy and his HbA1c increased to 9%.

**Fig. 4:**
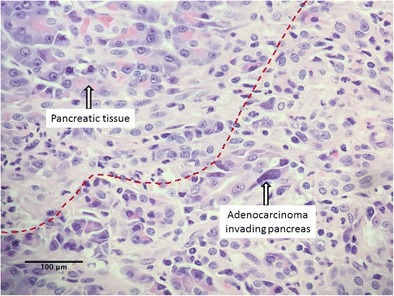
Invasive ampullary adenocarcinoma invades the pancreas

**Fig. 5:**
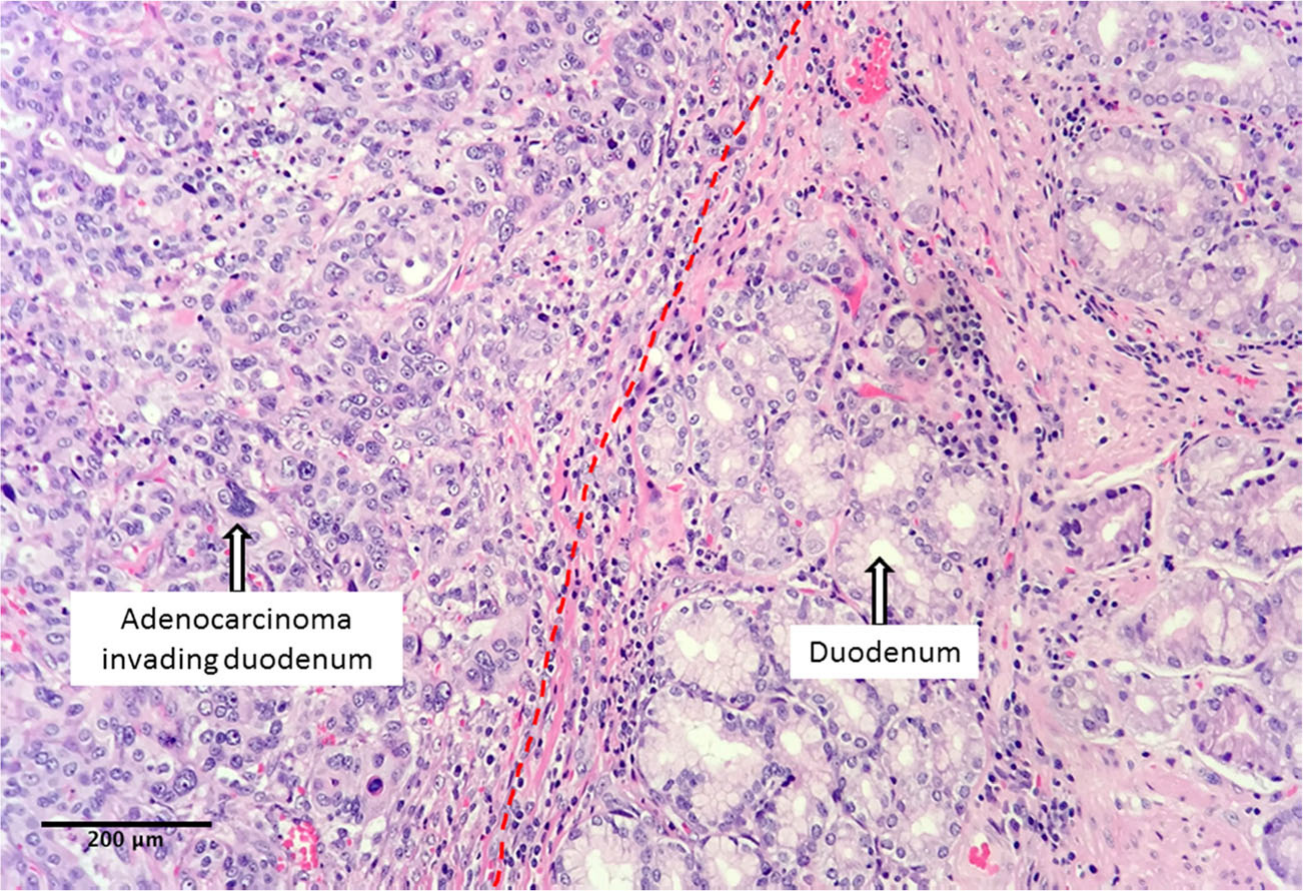
Invasive ampullary adenocarcinoma invades the duodenal wall

Final pathology of the lesion showed an invasive 2-cm ampullary adenocarcinoma, which was moderately differentiated (histologic grade G2) with negative margins; there was no involvement of the lymph nodes (0 of 29 nodes were positive; stage pT3N0M0) (Figs. 4 and 5). Multidisciplinary gastrointestinal tumor committee at our institution recommended postoperative adjuvant chemotherapy after patient’s full recovery from the surgery. CT performed 3 months after discharge was negative for recurrence. The patient’s recovery was prolonged due to his de-conditioned state pre- and after total pancreatectomy. He gained 3 lb of weight in 3 months, but still required hydrocodone/acetaminophen for pain. Six months after surgery, when he finally began to stabilize and thrive to the point that adjuvant chemotherapy might have been feasible, he developed metastatic disease—adenocarcinoma of the skin in the scar at the site of drain placement in the left upper abdomen. Immunohistochemistry demonstrated that the tumor was strongly and diffusely positive for CK7, focally positive for p63 and CDX2, and negative for CK5/6, confirming a gastrointestinal rather than skin origin of the tumor. A CT scan revealed local recurrence in the mid-abdomen and disseminated disease in the liver. Subsequently, the patient started chemotherapy treatment (cisplatin/gemcitabine). Soon after, a PET scan revealed metastases in patient’s lungs.

## Discussion

Adenocarcinoma of the ampulla of Vater is associated with early clinical symptoms of jaundice and abdominal pain. Pancreaticoduodenectomy is considered adequate (R0 resection) to obtain negative margins and achieve an appropriate lymph node dissection. Despite achieving an R0 resection, the survival rate at 2 and 5 years post-surgery remains 88.7 and 67.9%, respectively [8]. Total pancreatectomy does not improve outcomes and is associated with increased morbidity related to exocrine and endocrine pancreatic deficiency [9]. Therefore, islet autotransplantation with total pancreatectomy might be a vital option only in those instances where total pancreatectomy is offered due to additional factors that would render pancreaticoduodenectomy not feasible. It has been shown that auto-transplantation of islets significantly improve glucose control after the procedure, preventing brittle type of diabetes in 70% of patients [2]. Case reports of TPAIT in patients with periampullary cancer have confirmed such advantages, without any findings of disseminated neoplastic disease. However, the follow-up in those reports was short (3–30 months). Theoretically, a Whipple procedure removes all pancreatic tissue involved in the neoplastic process, so islet prep should not contain cancerous cells. Based on the above experience and rationale, a controlled study has been conducted in Italy, where patients with high risk for pancreatic fistula have been randomized intraoperatively to TPIAP or Whipple procedure [1]. The protocol includes several safety measures to minimize the risk of cancer cell contamination of the islet preparation. In a recent report from this center, no liver metastasis was found in 21 patients with median follow-up of 921 days [1].

Our patient had clear indication for TP due to the extensive damage of the entire pancreas by necrotizing inflammation and portal vein thrombosis. Additionally, he had a small, presumed early-stage ampullary cancer without lymph node involvement with no signs of metastatic disease. Therefore, we offered him IATx in order to improve his glucose control and quality of life. During the procedure, we obtained a low number of islet cells, with lower than usual viability, which was most likely related to the pancreatic damage caused by inflammation and ischemia prior to and during the surgery. Afterwards, the patient required TPN, which might have contributed to the poor glucose control (HbA1c of 9%), despite intensive insulin therapy. As soon as 6 months after the procedure, he developed local tumor recurrence in the abdominal cavity and disseminated disease in the liver and later in the lungs. However, the first presentation was a metastatic skin lesion with the features of gastrointestinal origin. This outcome was disappointing, especially considering that the disease seemed to be limited during initial presentation as well as based on the final pathology demonstrating negative margins and negative regional lymph nodes. We carefully followed the same safety measures as suggested in the “Milan Protocol” but didn’t perform islet purification since this would have further compromised the very low islet yield [1]. It is impossible to establish whether dissemination of the disease was triggered by islet autotransplantation or was a result of the natural biology of the tumor. It is also possible that tumor cells spread during acute necrotizing pancreatitis since multiple drainage procedures were required to assist patient in recovering from the acute process. Nevertheless, since our patient experienced a potentially fatal complication related to the metastatic disease, despite a favorable tumor stage at resection, we recognize that regardless of the tumor size and lymph node status, the decision to proceed with islet autotransplantation in patients with malignancy should be made with extreme caution. The patient should be informed about the potential complications, that cannot be calculated based on the tumor recurrence and dissemination. Although adjuvant chemotherapy could be beneficial, extending survival of patients with T3 and T4 tumor (median 32.2 vs 16.5) [10], we did not apply it due to the patient prolonged recovery and de-conditioning it is still uncertain whether it would have prevented the poor outcome if implemented.

In conclusion, our patient with an ampullary carcinoma despite the early clinical stage of pathology with negative lymph nodes and margins, developed metastatic disease 6 months after total pancreatectomy and islet autotransplantation. It is impossible to determine whether the disease recurrence resulted from natural biology of the tumor or it resulted from the infusion of the islet prep contaminated with cancer cells. Therefore, decision to apply islet autotransplantation in periampullary cancer patients remains controversial and should be considered with extreme caution.
